# Structural white matter changes in descending motor tracts correlate with improvements in motor impairment after undergoing a treatment course of tDCS and physical therapy

**DOI:** 10.3389/fnhum.2015.00229

**Published:** 2015-04-30

**Authors:** Xin Zheng, Gottfried Schlaug

**Affiliations:** Neuroimaging and Stroke Recovery Laboratory, Department of Neurology, Beth Israel Deaconess Medical Center and Harvard Medical SchoolBoston, MA, USA

**Keywords:** diffusion tensor imaging, motor recovery, rehabilitation, brain stimulation, tDCS, plasticity

## Abstract

Motor impairment after stroke has been related to the structural and functional integrity of corticospinal tracts including multisynaptic motor fibers and tracts such as the cortico-rubral-spinal and the cortico-tegmental-spinal tract. Furthermore, studies have shown that the concurrent use of transcranial direct current stimulation (tDCS) with peripheral sensorimotor activities can improve motor impairment. We examined microstructural effects of concurrent non-invasive bihemispheric stimulation and physical/occupational therapy for 10 days on the structural components of the CST as well as other descending motor tracts which will be referred to here as alternate motor fibers (aMF). In this pilot study, ten chronic patients with a uni-hemispheric stroke underwent Upper-Extremity Fugl-Meyer assessments (UE-FM) and diffusion tensor imaging (DTI) for determining diffusivity measures such as fractional anisotropy (FA) before and after treatment in a section of the CST and aMF that spanned between the lower end of the internal capsule (below each patient’s lesion) and the upper pons region on the affected and unaffected hemisphere. The treated group (tDCS + PT/OT) showed significant increases in the proportional UE-FM scores (+21%; SD 10%), while no significant changes were observed in an untreated comparison group. Significant increases in FA (+0.007; SD 0.0065) were found in the ipsilesional aMF in the treated group while no significant changes were found in the contralesional aMF, in either CST, or in any tracts in the untreated group. The FA changes in the ipsilesional aMF significantly correlated with the proportional change in the UE-FM (*r* = 0.65; *p* < 0.05). The increase in FA might indicate an increase in motor fiber alignment, myelination, and overall fiber integrity. Crossed and uncrossed fibers from multiple cortical regions might be one reason why the aMF fiber system showed more plastic structural changes that correlate with motor improvements than the CST.

## Introduction

Motor impairment after stroke has been related to the structural and functional integrity of descending corticospinal tracts (Canedo, 1997). Besides the corticospinal (CST) or pyramidal tract (PT), so-called alternate motor fibers (aMF) have been shown to play a role in modulating the degree of recovery after stroke (Lindenberg et al., 2010a, 2012a; Rüber et al., 2012). Based on animal studies, it has been hypothesized that the aMF is comprised of the cortico-rubro-spinal and cortico-reticulo-spinal systems (Lang and Schieber, 2003; Lindenberg et al., 2010a). In monkeys, upper extremity function was severely impaired after combined lesions to the contralateral PT and cortico-rubro-spinal tract, whereas animals with lesions solely to the PT showed considerable functional recovery (Lawrence and Kuypers, 1968). Similar observations have been made in humans after stroke (Lindenberg et al., 2010a, 2012a; Rüber et al., 2012).

Research over the last decade has also shown that stroke recovery can be facilitated by targeted non-invasive brain stimulation geared towards enhancing or diminishing local cortical excitability. Techniques such as transcranial direct current stimulation (tDCS) and repetitive transcranial magnetic stimulation (rTMS; Hummel et al., 2005; Mansur et al., 2005; Schlaug and Renga, 2008; Schlaug et al., 2008) have been used to up-regulate excitability in intact portions of ipsilesional (Hummel et al., 2005) or down-regulate excitability in contralesional motor cortex (Mansur et al., 2005). These approaches are based on neurophysiological studies, which indicate an imbalance of inter-hemispheric interactions after stroke resulting in disinhibition of the contralesional hemisphere and increased inhibition of the ipsilesional motor cortex (Murase et al., 2004). Single-session tDCS experiments in chronic stroke patients demonstrated that the uni-hemispheric modulation of motor cortex excitability yielded functional improvement of the affected upper extremity that outlasted the stimulation period (Fregni et al., 2005; Hummel et al., 2005; Celnik et al., 2009). In order to simultaneously target both components of this imbalance, a bi-hemispheric tDCS approach was proposed to target the motor cortex on the lesional hemisphere with anodal stimulation and the motor cortex on the contralesional hemisphere with cathodal stimulation (Schlaug and Renga, 2008). Vines et al. (2008) had shown that dual stimulation of both motor cortices lead to greater gains in learning motor sequences than unihemipsheric stimulation. More recently Waters-Metenier and colleagues (Waters-Metenier et al., 2014) demonstrated that tDCS effects generalized to untrained finger sequences of the trained hand as well as untrained hand making it an ideal adjunct to neurorehabilitative training regimens which require broad transfer to everyday tasks. Lindenberg et al. (2010b) showed that the dual hemispheric stimulation in chronic stroke patients daily for 5 days coupled with PT/OT lead to a 20.7% improvement in the UE-FM assessment and was significantly greater than what was seen in the group of patients that received sham stimulation with physical/occupational therapy (PT/OT) which only improved by 3.2%. The improvement in the experimental group was coupled with a stronger activation of the ipsilesional motor cortex and lesser activation of the contralesional motor cortex as determined in a functional MR imaging task when pre- and post-therapy images were compared. In a subsequent publication, Lindenberg and colleagues also found that bihemispheric stimulation coupled with PT/OT for 2 × 5 days, lead to more improvement than just 5 days in a row, however, the improvement in the second phase was less than what was seen in the first phase (Lindenberg et al., 2012b). Similar findings using a dual hemispheric stimulation approach coupled with Constraint-induced movement therapy (CIMT) were made by Bolognini and colleagues (Bolognini et al., 2011) who reported greater effects in the group of patients receiving real stimulation compared to the group that received CIMT with only sham stimulation (Bolognini et al., 2011).

These multi-session tDCS trials of stroke patients (Lindenberg et al., 2010b; Bolognini et al., 2011) as well as studies of healthy subjects (Vines et al., 2008; Waters-Metenier et al., 2014) suggest that stronger and longer-lasting effects can be obtained in multisession trials. Although it has been shown that either residual motor cortex activation or reaction after stroke (Lindenberg et al., 2010b) is important and that structural integrity of descending motor fibers (either the CST or aMF) determines whether or not a patient will make significant gains in any experimental motor recovery trial, it is not clear what functional or structural changes account for and underlie these longer-lasting behavioral improvements.

Studies over the last years have examined gray matter changes using voxel-based techniques as well as white matter changes particularly in regional diffusivity values in response to long-term training and skill acquisition paradigms. The fractional anisotropy (FA) is the most commonly used diffusivity measure. It is a normalized value between 0 and 1 and is a measure of the directional preference of water diffusivity. The higher the FA the more aligned fibers are in a particular voxel. Although voxel-based and deformation-based morphometric studies have shown increases in gray matter signal in training paradigms that spans years (Gaser and Schlaug, 2003; Hyde et al., 2009), the direction of FA changes in response to intense training and skill acquisition paradigms has been varied with increases and decreases being described. In general, higher FA values have been associated with more aligned fibers and possibly increased myelination, lower FA values might indicate less alignment of fibers and possibly axonal sprouting (Sidaros et al., 2008) and more branching (Hoeft et al., 2007). Two longitudinal studies have reported FA increases after individuals learned to juggle (Scholz et al., 2009), or underwent memory training (Engvig et al., 2012). Several studies have shown a decrease in FA in the ipsilesional posterior limb of the internal capsule (PLIC) (outside the stroke region) in subacute and chronic stroke patients (Yu et al., 2009; Puig et al., 2013). However, this decrease might reflect Wallerian Degeneration and not necessarily adaptation of tracts in response to natural or facilitated stroke recovery. In addition, Rüber et al. (2012) showed that compared to controls, chronic stroke patients had lower FA along the ipsilesional pyramidal tract and the aMF than healthy elderly controls, while clusters of higher FA were found bilaterally in the aMF tract in the vicinity of the red nuclei. We recently found FA decreases at nodal points of the arcuate fasciculus on the contralesional hemisphere in chronic aphasic patients undergoing an intense therapy period (Wan et al., 2014). As shown in the studies cited above, it is clear that white matter changes due to training or skill acquisition can manifest as FA changes—either increases or decreases—and the direction of this change may depend on the brain region or the type of training involved. As far as we can determine, no study has examined FA changes in descending motor tracts in response to an intense rehabilitation program in chronic stroke patients beyond any post-stroke adaptation process.

We used a pre-post design to examine microstructural white matter changes in descending motor tracts of the lesional and contralesional hemisphere in a group of ten chronic stroke patients with large left hemisphere lesions and at least moderate hemiparesis. This treated group underwent a 2 × 5-day course of combined noninvasive brain stimulation with tDCS (anodal electrode over motor cortex of the lesional hemisphere and cathodal electrode over motor cortex of the contralesional hemisphere) combined with a combination of physical and occupational therapy (for details, see Lindenberg et al., 2010b, 2012b). Diffusion tensor imaging (DTI) scanning as well as motor assessments was administered before and after the experimental treatment phase. A separate group of patients who had undergone DTI scanning and motor assessments over a similar time frame was used as a comparison group (untreated group). The main goal of our study was to determine whether a short-term experimental intervention consisting of non-invasive brain-stimulation using tDCS for 30 min in combination with PT/OT for 60 min for 2 × 5 days in a row could lead to changes in DTI-derived measures of major descending motor tracts.

## Methods

### Participants

The treated group (Age: 57.5 years, SD 12.9; Time Post-Stroke to Enrollment: 19.2 months, SD 17.4; Scan Interval: 34.7 days, SD 15.0; wCST-Lesion Load: 5.4 cc, SD 5.3) consisted of ten stroke patients (≥4 months after their first and only unihemisphere stroke) who participated in an experimental study consisting of a combination of dual transcranial direct current stimulation (tDCS) for 30 min while simultaneously receiving physical/occupational therapy (PT/OT) for 60 min (for details of the intervention, see Lindenberg et al., 2010b, 2012b) and had DTI studies done before and after the intervention. This group was contrasted to an untreated group of ten patients (Age: 58.5 years, SD 9.4; Time Post-Stroke to Enrollment: 25.9 months, SD 30.2; Scan Interval: 33.9 days; SD 11.3; wCST-Lesion Load: 8.2 cc, SD 4.9), matched for age, time post-stroke-to-enrollment, and between-scan interval who had not undergone any experimental intervention, but were scanned twice with DTI while waiting to be enrolled in a treatment study. All patients underwent motor assessments including the Upper Extremity Fugl-Meyer assessments (UE-FM) before and after the intervention.

In order to create canonical tracts of CST and aMF, we used data from twelve healthy elderly subjects (9 male; mean age: 56.5 SD 14.8 years) who were scanned once.

All subjects gave their written informed consent following protocol approved by the Committee of Clinical Investigations and Internal Review Board at Beth Israel Deaconess Medical Center.

### Treatment and Behavioral Assessment

The treatment consisted of 10 sessions of tDCS (for 30 min/day) in conjunction with PT/OT (for 60 min/day) over a 2–3 week span. PT and OT techniques included functional motor tasks of the affected arm and hand to promote sensorimotor integration, coordination of movement, and goal-directed activities of practical relevance for the patient. It was left to the physical therapist to determine the motor activities/tasks that would be most helpful for a particular patient. Direct current was delivered through 2 saline-soaked surface gel-sponge electrodes (electrode area was 16.3 cm^2^) using a Phoresor II Auto stimulator (IOMED, Salt Lake City, Utah). The stimulation consisted of 30 min of 1.5 mA direct current with the anode placed over the ipsilesional motor cortex and the cathode over the contralesional motor cortex. Stimulation sites were identified using the international 10–20 EEG electrode system.

Each patient underwent the Upper-Extremity Fugl-Meyer Assessment (UE-FM) before and after treatment. The UE-FM assessment is a standardized and validated 30-item motor impairment assessment frequently used in stroke rehabilitation (Fugl-Meyer et al., 1975) with excellent inter- and intra-rater reliability (Duncan et al., 1983) with higher scores (max score is 66) reflecting more complete functionary recovery. The patients in the treated group underwent UE-FM assessment before and after treatment, while the untreated group received two UE-FM assessments at a similar time interval apart. Some patients had more than one UE-FM assessment done at each time point. Assessments were averaged if that happened (see Table 1).

**Table 1:** **Biographical and and lesion information for patients in the treated and untreated groups**.

Treated group	Age (years)	Time post-stroke to enrollment (months)	Lesioned hemisphere	Lesion volume (cc)	wCST-lesion load (cc)	UE-FM baseline	UE-FM after therapy	Time interval between scans (days)
1	77	4	L	3.9	2.3	19	26.5	12
2	70	46	L	252.4	14.3	32.5	40.7	28
3	51	6	R	68.5	15.5	37	41	22
4	49	16	R	7.0	2.2	40.5	50	39
5	35	6	R	8.1	6.3	18	22	32
6	71	13	L	5.2	2.6	52.5	58	60
7	51	39	R	39.2	2.8	48	52	54
8	51	16	R	11.9	1.9	32	39.5	24
9	52	5	R	206.0	0.3	27	37.7	30
10	63	8	L	10.3	6.3	20	20.5	46
**Untreated group**	**Age (years)**	**Time post-stroke to enrollment (months)**	**Lesioned hemisphere**	**Lesion volume (cc)**	**wCST-lesion load (cc)**	**UE-FM baseline**	**UE-FM after therapy**	**Time interval between scans (days)**
1	63	15	L	226.5	20.1	12	13	46
2	51	12	L	63.5	1.8	63	63	34
3	61	26	L	273.7	16.3	18	17	34
4	65	110	L	188.7	11.5	14	14	27
5	66	10	L	155.1	3.3	8	8	56
6	35	9	L	198.8	12.3	19	21	33
7	63	27	R	77.2	7.1	20.5	18	14
8	59	17	L	108.3	10.6	18	18	35
9	56	16	L	196.9	3.0	65	65	26
10	58	17	L	75.8	8.8	11	11	34

### Image Acquisition

All patients underwent MRI scanning using a 3-Tesla General Electric (Fairfield, CT) scanner. The treated group was scanned before and after therapy, while the untreated group was scanned two times with similar time intervals between scans as the treated group. The group of healthy elderly subjects used to generate the canonical CST and aMF were scanned only once.

All subjects underwent anatomical scans, which were acquired using high-resolution strongly T1-weighted Magnetization Prepared Rapid Acquisition Gradient Echo (MPRAGE) sequence (voxel size = 0.93 × 0.93 × 1.5 mm). Two types of DTIs protocols were used. Seven patients in each group (treated and untreated) were acquired using a single-shot, spin-echo, echo-planar imaging sequence (TE = 86.9 ms, TR = 10,000 ms, FOV = 240 mm, slice thickness = 5 mm, resolution: 1.87 × 1.87 × 5.0 mm, no skip, NEX = 1, axial acquisition, 25 non-collinear directions with *b*-value = 1000 s/mm, 1 image with *b*-value = 0 s/mm). Three patients in each group were acquired using a single-shot, spin-echo, echo-planar imaging sequence (TE = 86.9 ms, TR = 10,000 ms, FOV = 240 mm, slice thickness = 2.5 mm, resolution: 2.5 × 2.5 × 2.5 mm, no skip, NEX = 1, axial acquisition, 30 non-collinear directions with *b*-value = 1000 s/mm, 6 images with *b*-value = 0 s/mm).

### Processing of Diffusion Tensor Imaging Data

The diffusion data were processed using FSL (4.1.4).^1^ Preprocessing steps included correction for eddy current effects, skull stripping, head motion correction with affine multi-scale two-dimensional registration, as well as estimation and fitting of a diffusion tensor model at each voxel using *DTIFIT* to calculate the lambda values for each principle eigenvector and FA.

For the patients, we registered the FA image of the first time point to the FMRIB standard FA template using linear and non-linear algorithms (*FLIRT* and *FNIRT*), with the aid of lesion masks. The FA image from the second time point was then registered to the normalized FA image from the first time point. All FA images with a left hemisphere lesion were flipped such that all images had the stroke lesion in the right hemisphere. The FA images of the healthy control subjects were normalized directly to the FMRIB standard FA template. *BEDPOSTX* was conducted on these images in preparation for tractography.

### Tractography

Tractography of the aMF and CST were outlined in a previous publication (Lindenberg et al., 2010a) and was only done for the 12 healthy elderly control subjects to generate the canonical tracts for CST and aMF. A single slice seed region was drawn at the pontine level (approximate Talairach *z* = −24) as a narrow ROI that included only the anterior part of the pons (for the CST) and similarly a single slice ROI was drawn in the posterior part of the pons (for the aMF). Additional ROIs were drawn in the PLIC and the white matter underlying the posterior part of the precentral gyrus. Exclusion ROIs were drawn on the superior and medial cerebellar peduncle to exclude fibers to the cerebellum, as well as the middle sagittal region covering the brain stem and corpus callosum to exclude trans-hemispheric fibers. *Probtrackx*^2^ was run to track fibers from either of the two pons ROIs as the seeding region. Tracts were normalized to the SPM5 T2 template from SPM5 (Wellcome, Department of Neurology, London, UK) implemented in MATLAB (The Mathworks, Inc., Natick, MA), which was achieved by normalizing the DWI image to the SPM5 T2-template, and then applying the normalization parameter to each CST tract. A 50th percentile threshold was applied to each CST fiber as well as the aMF, and then the twelve tracts were each binarized and summed to create the summed CST and aMF. This summed CST and aMF was then thresholded again such that only voxels where at least six subjects (50%) have a tract was included to create our canonical CST and aMF.

Only the portion of these canonical tracts between the lower end of the internal capsule (approximately Tailarach *z* = −21 to *z* = 4; see Figure 2)—which was below the lesion of each patient—and the upper pons were used to determine diffusivity measures such as the FA as well as the axial, radial and mean diffusivity (MD) values.

### Processing of Anatomical Imaging Data

The anatomical (T1-weighted) images of each patient were normalized to FSL’s skull-included T1 template. Lesion maps were drawn on the normalized T1-weighted images by an experienced neurologist using T1- and coregistered T2-weighted images as a reference. The lesion maps were then overlapped with the probabilistic CST (for details see Zhu et al., 2010) in order to calculate a weighted CST lesion load. The weighted CST lesion load is a probabilistic quantification that represents the degree of damage to the CST, putting more weight to regions that are present in more subjects as well as putting more weight to areas of higher fiber concentration (smaller width = higher concentration) such as when the CST is concentrated in the internal capsule.

### Statistical Analysis

All left-hemisphere lesions were flipped to the right hemisphere for analysis. FA and other diffusivity measures were extracted from the CST and aMF templates on the lesional and contralesional hemisphere in both the treated and untreated groups at both time points. Paired *t*-tests were then conducted to test for pre- vs. post-treatment differences in the treated and untreated groups. A Bonferroni correction was done to correct for alpha inflation. Thus, only *p* < 0.0125 (*p* = 0.05/4) is considered to be significant. Mean FA values from tracts that were found to be significantly changed after treatment were correlated with proportional UE-FM change scores.

## Results

**Figure 1:**
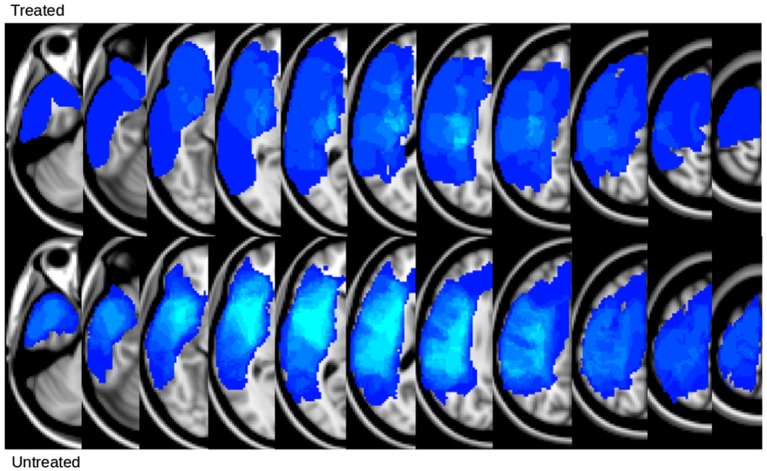
**Lesion density map of treated and untreated groups**. Lighter blue indicates voxels with greater lesion overlap across patients.

**Figure 2:**
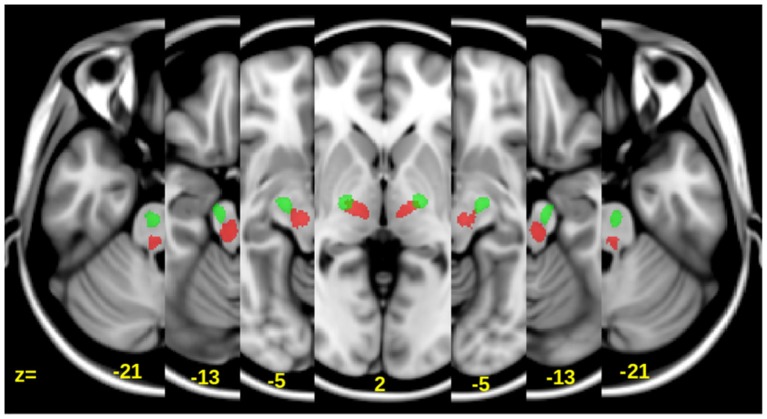
**Axial slices showing the location of the CST (Green) and alternate motor fibers (aMF) (Red) overlaid onto a T1 template**. The *z*-coordinates are in Tailarach space.

In Figure 1, we show the lesion density maps of the treated and untreated groups. There was a trend for higher lesion volumes and wCST-lesion loads (wCST-LL) in the untreated group (Table 1), but wCST-LL was not significantly different between groups (*p* > 0.1). Otherwise, both groups were well matched with regard to age, time post-stroke-to-enrollment, and between-scan interval. The wCST-LL is a novel measure that combines lesion volume and lesion location by calculating a weighted overlap region between each patient’s lesion and the canonical corticospinal tract as it descends from the cortex through the internal capsule into the brainstem. As one can see from Figure 1, the density maps overlap with a large portion of the middle cerebral artery territory and striatocapsular region, including significant portions of the corticospinal tract from the cortex to the internal capsule. In order to not confound our analysis of tract changes by the variable lesion overlap with the descending motor tracts, we chose to evaluate only the portion of the CST and the aMF from the lower end of the internal capsule on downwards to the upper portion of the pons. Although the CST and aMF overlap to some degree in the internal capsule region and further upstream, from the internal capsule on downwards, these two tracts start to separate more and take a different course through the cerebral peduncle and brainstem (Figure 2).

**Figure 3:**
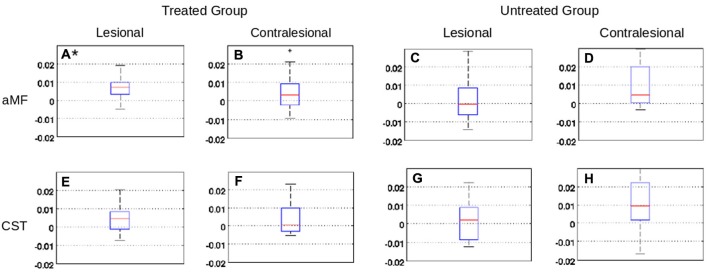
**Graphs showing fractional anisotropy (FA) changes (± standard deviation) in the CST and aMF ROIs, in the treated and untreated group, on both hemispheres**. Only the FA extracted from the aMF ROI on the lesion side of the treated group showed a significant (*p* ≤ 0.01, Bonferroni corrected) increase in FA (see panel A*). Remaining regional values in the treated group **(B,E,F)** or regional values in the untreated group **(C,D,G,H)** did not show significant changes.

**Figure 4:**
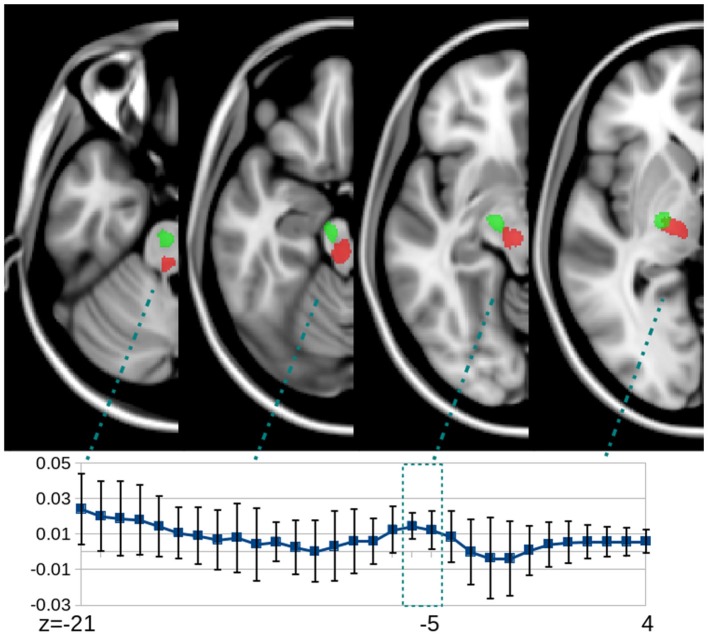
**Slice-by-slice cross-sectional plot of the FA change in the aMF ROI (red color) on the lesional side of the treated group (mean and SD)**. ROIs (red = aMF; green = CST) are superimposed onto a standardized T1-weighted image. Most pronounced and most consistent increases in FA across the entire group can be seen at the pontine level (z = −21, −20) and in a location in the vicinity of the red nucleus which typically can be found between z = 0 to −8, Talairach coordinates.

The mean FA changes in the CST and aMF on the lesional as well as contralesional side were calculated. Significant increases in FA (+0.0070; SD 0.0065, *p* < 0.01) were only found in the ipsilesional aMF in the treated group while no significant changes (all *p* > 0.2) were found in the contralesional aMF, CST in either hemisphere, and in the untreated group (see also Figure 3). Two subregions of the aMF stood out: the mid to upper portion of the pons and the white matter surrounding the red nucleus showed the most pronounced and most consistent FA increases across all subject (Figure 4). None of the other tracts showed any consistent and significant changes. Even when we considered CST lesion load in the analysis, we could not detect any significant FA changes in the CST that was inversely related to CST lesion load (*p* > 0.2). The increase in FA in the ipsilesional aMF was mainly due to an increase in axial diffusivity (+1.07, SD 0.26), although this did not become statistically significant (*p* > 0.1). We did not detect any significant changes or even any non-significant trends in radial diffusivity (RD) or in MD in any of the tracts (*p* > 0.2).

**Figure 5:**
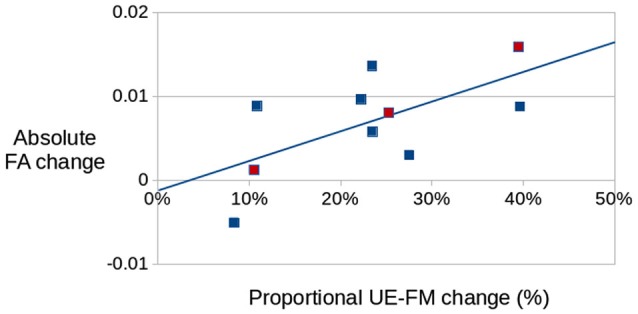
**Correlation between changes in FA (obtained from the aMF on the lesional hemisphere) and proportional changes in UE-FM in the treated group (*r* = 0.65; *p* < 0.05)**. Red dots are those from the diffusion tensor imaging (DTI) scan that used 30 directions. No differences were seen between the DTI sequences that used 25 directions and the one that used 30 directions.

The FA changes in this portion of the aMF were also significantly correlated (Figure 5) with proportional change in UE-FM (*r* = 0.65; *p* < 0.05).

## Discussion

In this study we found a significant increase in FA in the portion of the ipsilesional aMF that spans between the inferior internal capsule and the upper pons. This increase in FA correlated with the proportional change in UE-FM comparing pre- with post-intervention assessments. The most significant and most consistent FA change across subjects was seen in the portion of the aMF in the mid to upper pons region and in the white matter in the vicinity of the red nucleus. None of the other tracts showed any consistent or significant changes across subjects in the treated group. No significant changes were seen in the control group.

The red nucleus and its surrounding white matter has been shown to have alterations of FA values when compared to normal elderly controls in a cross-sectional analysis of chronic stroke patients (Rüber et al., 2012). This could indicate that remodeling of this fiber system occurs also naturally after stroke, possibly indicating a compensatory role of aMF and its rubral relay station for motor recovery after damage to the corticospinal system. Similarly, Takenobu and colleagues (Takenobu et al., 2014) found significantly increased FA values in the vicinity of the red nucleus and dorsal pons in the ipsi lesional side at 3 months after stroke in 10 patients with subcortical strokes involving the internal capsule. While the FA increases in Takenobu’s study (Takenobu et al., 2014) mainly reflect changes in response to natural recovery, the FA increases in our experimental treatment study reflect treatment-induced changes. Both studies showed that the increases in FA were correlated with motor improvement.

Experimental animal studies have demonstrated that recovery of motor function after CST lesions can be mediated by the rubro-spinal tract (Lawrence and Kuypers, 1968) and are associated with a change in the synaptic organization of efferent neurons in the red nucleus (Belhaj-Saïf and Cheney, 2000). Similar observations have been made in humans after stroke. Despite severe damage of the pyramidal tract, motor evoked potentials from ipsilesional motor cortex could be elicited in the affected limb of chronic stroke patients (Fries et al., 1991) and patients were able to independently control individual fingers of their affected hands (Lang and Schieber, 2003). Based on these studies and the findings of our current study, we postulate that the aMF might assume a compensatory function in stroke patients with severe CST damage (Fries et al., 1991; Lang and Schieber, 2003; Lindenberg et al., 2010a; Rüber et al., 2012; Takenobu et al., 2014). Additional evidence comes from a DTI-study reporting higher FA values in ROIs in the ipsilesional red nucleus in subacute stroke patients as compared to healthy controls (Yeo and Jang, 2010). Furthermore, we found in a previous cross-sectional study FA alterations in bilateral red nuclei and strong correlations with measures of motor function (Rüber et al., 2012). Event though there are some hints in the literature that the rubrospinal tract might show changes on both sides (Rüber et al., 2012), we did not find any evidence for any significant changes in the contralesional CST as has been reported in other studies (Schaechter et al., 2009) or in the contralesional aMF in our group of patients.

After a stroke that affects the CST partially or completely, there is usually a pronounced decrease of FA values in the affected CST distal to the lesion (Werring et al., 2000; Thomalla et al., 2004; Takenobu et al., 2014), more than what is found in aMF (Rüber et al., 2012), indicating that the CST might be more prone to Wallerian degeneration after a stroke that affects the motor system than multisynaptic fibers constituting the aMF which might draw fibers from more widespread and possibly bihemispheric regions and might therefore be less affected by a unihemispheric stroke (Lindenberg et al., 2010a; Rüber et al., 2012). Furthermore, there is cross-talk at the rubral and tegmental level and the rubral as well as tegmental tracts even on the lesional side might contain crossed and uncrossed fibers (Canedo, 1997; Rüber et al., 2012). Interestingly, the nodal points of FA increases in the pons and in the vicinity of the red nucleus in our study also correlate with observed increased microglial activity in the subacute to chronic stroke phase (Thiel and Heiss, 2011) possibly indicating continued remodeling of tracts after a stroke which seems to be more concentrated in some areas than others over the course of the CST and aMF.

Rüber et al. (2012) also showed an increased probabilistic connectivity in chronic stroke patients arising from the peri-rubral region which may reflect structural adaptations of the red nucleus caused by compensatory input to this relay station in the upper midbrain. Similar findings were reported in neonatal rats with lesions to the pyramidal tract as a result of lesion-induced sprouting (Z’Graggen et al., 2000). This increased connectivity of the rubral system might indicate that fibers from more widespread cortical regions, possibly from both hemispheres, might descend onto the red nuclei on both sides. This could explain that this system could show plastic changes after a stroke, since it might contain fibers from unaffected parts of the brain.

Several studies in healthy individuals have reported training-induced modifications of white matter architecture. While some showed training-related FA increases (Scholz et al., 2009; Engvig et al., 2012), others reported FA decreases (Elmer et al., 2011; Halwani et al., 2011; Wan et al., 2014). This discrepancy may reflect the different mechanisms by which different brain regions can remodel. Variations in FA across and within individuals over time can be influenced by factors such as fiber density, axon diameter, myelination, axon collateral sprouting, cell membrane density, and fiber coherence (Song et al., 2002, 2003; Budde et al., 2007; Hoeft et al., 2007; Sidaros et al., 2008). In general, higher FA values have been associated with more aligned fibers and possibly increased myelination, lower FA values might indicate less alignment of fibers and possibly axonal sprouting (Sidaros et al., 2008) and more branching (Hoeft et al., 2007). In a previous intense experimental treatment study in chronic aphasic patients (Wan et al., 2014), we were able to show that FA decreases over time occurred more in closer proximity to the cortex, reflecting a more complex and less aligned fiber tract, possibly indicating axonal sprouting, when the connection between distal cortical regions becomes important for the therapy success. Similar to the current study, we found the change in FA after treatment was associated with a greater improvement in speech fluency.

There are a few limitations of our study. First, we used a relatively short DTI sequence (<5 min) in 7 of 10 patients in each of the two groups. This sequence was chosen to minimize movement artifacts in our group of moderate to severely impaired patients. However, this diffusion sequence had non-isotropic voxels and 25 diffusion directions. Acquiring higher resolution images with isotropic voxels could improve the accuracy of parameter estimation in the DTI analysis. Nonetheless, any systematic errors associated with our DTI sequence should be equally evident across all tracts and both groups. Second, this pilot study has a relatively small sample of experimentally treated patients which might limit generalizability of our results. However, our interpretation correlating rubro-spinal tract microstructural changes with motor recovery may be extrapolated to other stroke patients and other tracts. Third, we cannot determine whether the FA effects in the aMF are due to tDCS alone, physical/occupational therapy alone, or a combination of both as we suggest in this paper. Control groups of chronic stroke patients in previous studies who received any kind of peripheral sensorimotor activities to stimulate recovery, showed relatively little change (e.g., only 3.2% in UE-FM in the group who received sham-tDCS and PT/OT in Lindenberg et al. (2010b) and it would be hard to believe that this little change compared to the change induced by the combined intervention, could be correlated with the structural change observed in this study. Furthermore, many studies have now been published showing effects of combined brain stimulation with peripheral sensorimotor activities and there is experimental evidence from animal work that combined stimulation (central and peripheral) increases synaptic plasticity (Fritsch et al., 2010). Fourth, one could potentially make the argument that the lack of an effect in the affected CST was due to large lesions affecting the CST on the lesional hemisphere. However, we did not find a clear pattern of an inverse relationship between wCST-LL and FA changes. Indeed the minimal CST changes if present at all were either negative or positive across subjects would go in both directions and did not follow any particular pattern. Nevertheless, large studies will probably be necessary to examine this in more detail.

In summary, we interpret the observed FA changes in the pons and in particularly in the vicinity of the ipsilesional red nucleus in the experimentally treated chronic stroke patients as a result of plastic remodeling of a polysynaptic tract that receives more widespread cortical input than the CST and has crossed and uncrossed fibers as has been shown in animal and human studies. The strong correlation of local FA values with UE-FM scores suggests that the observed diffusivity alterations are functionally meaningful. We hypothesize that the cortico-rubral and cortico-tegmental tracts which are making up the aMF could be particularly sensitive and responsive to experimental treatments that modulate cortical excitability in both sides of the brain involving primary and non-primary motor regions and could play a more prominent role in post-stroke recovery than has been assumed so far.

## Conflict of Interest Statement

The authors declare that the research was conducted in the absence of any commercial or financial relationships that could be construed as a potential conflict of interest.

## Footnotes

^1^http://www.fmrib.ox.ac.uk/fsl

^2^http://www.fmrib.ox.ac.uk/fsl/fdt/fdt_probtrackx.html
